# Development of a nomogram for predicting wall irregular pulsation and risk stratification in intracranial aneurysms

**DOI:** 10.3389/fneur.2026.1913037

**Published:** 2026-09-28

**Authors:** Jinjin Liu, Jiafeng Zhou, Jianjian Zhang, Xiao Li, Olivia Li, Huilin Zhao, Yisong Zhang, Bing Zhao

**Affiliations:** 1 Department of Radiology, The First Affiliated Hospital of Wenzhou Medical University, Wenzhou, Zhejiang, China; 2 Department of Neurosurgery, Renji Hospital Shanghai Jiaotong University School of Medicine, Shanghai, Zhejiang, China; 3 Department of Radiology, Renji Hospital Shanghai Jiaotong University School of Medicine, Shanghai, China; 4 Cornell University, Ithaca, NY, United States; 5 Department of Neurosurgery, Pudong New Area People's Hospital Affiliated to Shanghai Jiao Tong University School of Medicine, Shanghai, China

**Keywords:** aneurysm wall irregular pulsation, four-dimensional computed tomography angiography, intracranial aneurysm, nomogram, rupture risk

## Abstract

**Objective:**

Accurate rupture risk stratification for unruptured intracranial aneurysms (UIAs) presents clinical challenges. Aneurysm wall irregular pulsation is an imaging biomarker of aneurysm vulnerability. This study aims to develop a nomogram for predicting wall irregular pulsation and risk stratification in intracranial aneurysms.

**Methods:**

This was a consecutive cross-sectional observational study with prospective data collection for UIAs between 2017 and 2020. The irregular pulsation was defined as sequential morphological alterations observed in three or more consecutive phases by on four-dimensional CT angiography (4D-CTA). A generalized estimating equation (GEE) method identified independent predictors of the presence of wall irregular pulsation. A logistic regression-based predictive model was developed. Model performance was evaluated using receiver operating characteristic analysis.

**Results:**

We included 202 patients with 262 unruptured aneurysms. Multivariable analysis revealed four independent predictors of the presence of wall irregular pulsation: aneurysm location at ACA/PCom/Posterior (OR: 2.17, 95% CI 1.21–3.88; *p* = 0.0099), presence of daughter sac (OR: 5.15, 95% CI 1.91–13.86; *p* = 0.001), aspect ratio (OR: 1.80, 95% CI 1.09–2.99; *p* = 0.022), and neck size (OR: 1.23, 95% CI 1.02–1.48; *p* = 0.030). The developed model demonstrated good discrimination, achieving a sensitivity of 88.5%, specificity of 71.2%, overall accuracy of 76.9%, and an area under the curve (AUC) of 0.808 (95% CI, 0.704–0.913). Further validation using an independent cohort yielded comparable performance, with a sensitivity of 76.9%, specificity of 88.9%, overall accuracy of 81.8%, and an AUC of 0.855 (95% CI, 0.694–1.000). A nomogram was constructed to visualize the model.

**Conclusion:**

This study presents a logistic regression-based model for predicting aneurysm wall irregular pulsation. The nomogram visually assesses individualized risks, demonstrating potential for future clinical implementation in bedside decision-making.

## 1 Introduction

The advancement of modern imaging modalities, including computed tomography angiography (CTA) and magnetic resonance angiography, has significantly increased the detection of unruptured intracranial aneurysms (UIAs) (1). The clinical management of UIAs presents a persistent therapeutic dilemma because of a relatively low risk of rupture compared with treatment risks (2). However, aneurysm rupture leading to subarachnoid hemorrhage (SAH) has devastating consequences. Thus, accurate rupture risk stratification of UIAs is crucial for optimizing clinical decision-making. In recent years, irregular pulsation of the aneurysm wall has been identified as a potential biomarker of aneurysm vulnerability.

Morphological changes frequently develop at sites of irregular pulsation during follow-up, and this zone typically corresponds to the bleeding site in most ruptured aneurysms (3, 4). Ferrari et al. (5) established the reliability of four-dimensional CTA (4D-CTA) in characterizing both dynamic and structural aneurysm wall features, with observed macroscopic features of aneurysm wall, such as pulsation in particular, corresponding to the dark-reddish thinner wall. Our previous study has shown that aneurysm irregular pulsation, identified by 4D-CTA, is significantly associated with symptomatic and ruptured aneurysms (6). Furthermore, combining irregular pulsation with vessel wall MRI enhancement patterns yields optimal diagnostic accuracy for identifying symptomatic aneurysms (7). These findings suggest that irregular pulsation may contribute to aneurysm rupture or growth, and can serve as a marker for identifying high-risk UIAs.

With the rapid advancement of artificial intelligence, advanced computational and image-processing algorithms have been extensively applied in medical imaging, demonstrating clinical value in aneurysm segmentation, diagnostic evaluation, and prognosis assessment (8–12).

In the present study, we utilized 4D-CTA to identify irregular pulsation, and developed a logistic regression-based model incorporating available clinical parameters to predict the presence of wall irregular pulsation in UIAs.

## 2 Materials and methods

### 2.1 Study design

The study received approval from our institution, and written informed consent was acquired from all participants or their legal representatives. This was a prospective, consecutive cross-sectional observational study of intracranial aneurysms confirmed by 4D-CTA and digital subtraction angiography, conducted between October 2017 and January 2020 (13). We collected clinical data prospectively, including demographic characteristics, medical history (previous subarachnoid hemorrhage), smoking status, alcohol use, hypertension, hyperlipidemia, and diabetes mellitus. An independent external cohort consisted of 22 aneurysms from 20 patients, collected between January 2025 and June 2025 at Renji Hospital, Shanghai Jiaotong University School of Medicine, for validation. Comparison of baseline characteristics between development and validation cohorts is provided in the Supplementary Table 1.

In this study, we included participants if they were between 18 and 85 years of age and had at least one UIA. Exclusion criteria were as follows: ruptured aneurysms, current pregnancy or lactation; severe concomitant conditions, including malignant tumors and hematologic disorders; systemic comorbidities such as renal impairment or recent acute myocardial infarction; secondary aneurysms resulting from trauma or infection; fusiform or dissecting morphology; and inadequate image quality.

### 2.2 Protocol of 4D-CTA and aneurysm wall irregular pulsation

We conducted electrocardiographically synchronized 4D-CTA using a 320-detector row CT system (Aquilion One Vision, Canon Medical Systems, Otawara, Japan). The imaging procedure began with a non-contrast cranial CT scan performed at 120 kV with automated tube current modulation. This was followed by intravenous injection of 60 mL of iodinated contrast agent (iomeprol 400 mg/mL, Iomepron; Bracco, Milan, Italy) at a rate of 3–4 mL/s. A bolus-tracking technique was used, with manual triggering upon contrast opacification of the middle cerebral artery.

Scanning parameters included a tube voltage of 120 kVp and a current of 230 mA, a gantry rotation time of 275 ms, a slice thickness of 0.5 mm, and a longitudinal coverage of 16 cm, yielding a voxel resolution of 0.31 × 0.31 × 0.5 mm^3^. A continuous volumetric scan covering one full R-R interval was performed, producing 20 phase datasets, each corresponding to a 5% step within the cardiac cycle.

Image post-processing was carried out using the scanner’s integrated reconstruction software, which features the Adaptive Iterative Dose Reduction 3D (AIDR 3D) algorithm with an FC43 kernel. This generated a 20-phase image series reconstructed at 0.5 mm slice thickness and interval.

Aneurysm irregular pulsation was identified as sequential morphological changes—such as transient convex protrusions, concave deformations, or focal expansions of the aneurysm wall—observed across three or more consecutive phases in the 4D-CTA series. A representative example is illustrated in Figure 1. Two experienced neuroradiologists independently assessed irregular pulsation, with any discrepancies resolved through consensus review with a third neuroradiologist.

**Figure 1:**
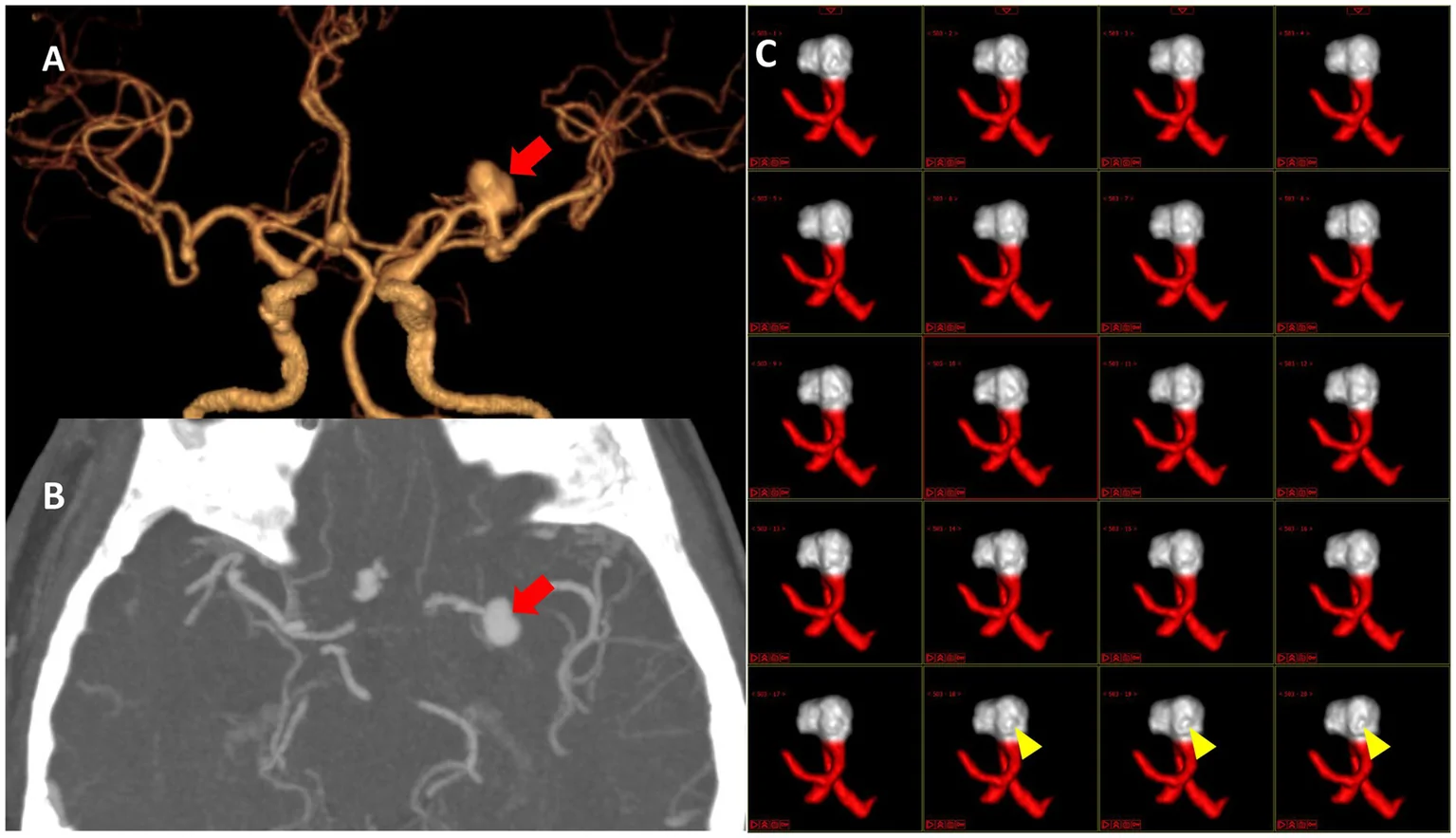
A representative case of irregular pulsation. A 54-year-old female patient has an aneurysm in the M1 segment of the left middle cerebral artery, with a size of 9.2 mm. **(A)** Digital subtraction angiography; **(B)** CTA maximum intensity projection (MIP); and **(C)** Irregular pulsation is observed in the 18th to 20th frame of 4D-CTA (yellow arrow).

### 2.3 Aneurysm morphologic measurements

We quantified a series of morphometric characteristics of the aneurysms, incorporating key dimensional and angular parameters (13). These included aneurysm size, neck diameter, perpendicular height, vessel size, and aneurysm, vessel, and flow angles. A schematic representation of these measurements is provided in Figure 2.

**Figure 2:**
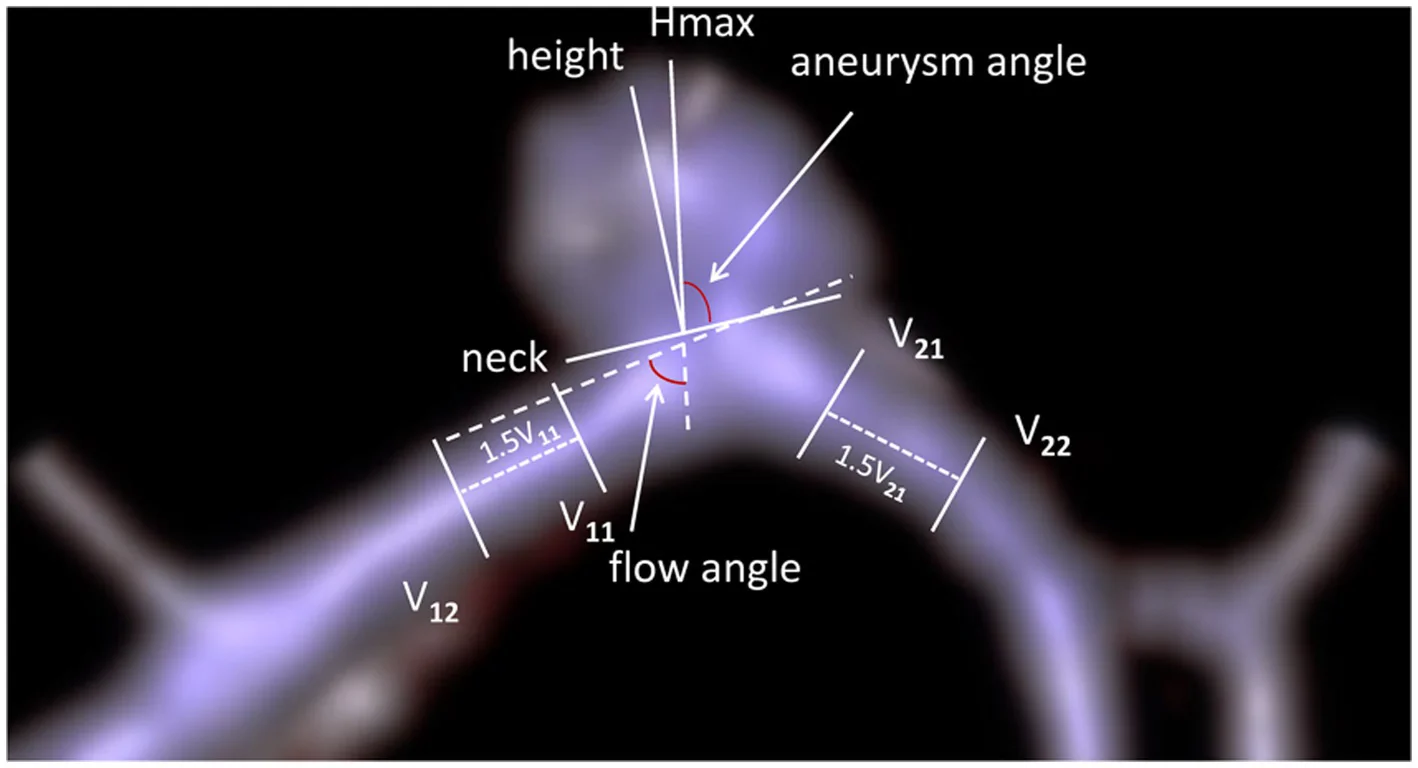
Illustrations of morphological measurements.

Vessel size was computed as the mean of the cross-sectional diameters measured at two locations: immediately adjacent to the aneurysm neck (Vi1) and at a distance equal to 1.5 times Vi1 distal to the neck (Vi2), expressed as Dvi = (Vi1 + Vi2)/2, where i denotes one of three possible measuring positions. The maximum aneurysm height was defined as the largest cross-sectional diameter of the aneurysm sac. The perpendicular height represented the shortest distance from the midpoint of the neck plane to the apex of the dome.

For angular parameters, the aneurysm angle was measured as the inclination between the plane of the aneurysm neck and the vector corresponding to the maximum height. The vessel angle reflected the divergence between the parent artery’s blood flow direction and the plane of the aneurysm neck. The flow angle was characterized as the angle between the direction of flow in the parent vessel and the axis of maximum aneurysm height. Additionally, two derived ratios were calculated: the size ratio, reflecting the quotient of the maximum aneurysm height and the vessel size, and the aspect ratio, defined as the perpendicular height divided by the neck size.

Aneurysm location was classified into three categories according to the PHASES score: (1) internal carotid artery (ICA), (2) middle cerebral artery (MCA), and (3) a combined category comprising the anterior cerebral arteries (ACA, including the anterior cerebral artery, anterior communicating artery, and pericallosal artery), the posterior communicating artery (PCom), and the posterior circulation (including the vertebral artery, basilar artery, cerebellar arteries, and posterior cerebral artery) (14).

### 2.4 Model development

We developed a logistic regression-based model to predict the potential risk of irregular pulsation. Model construction employed patient-level stratified random sampling to partition the dataset into training (80%) and test (20%) sets. This approach ensured that all aneurysms from the same patient were assigned to the same partition, preserving the clustered nature of the data. Variable selection was guided by clinical relevance/experience, and results from univariate generalized estimating equation (GEE) analysis. Although prior SAH did not reach statistical significance in the univariate analysis, we decided to keep it in the model anyway—mainly because it’s a key element of both the PHASES and ELAPSS scores (14, 15), which are widely used for rupture risk assessment. The final logistic regression model was fitted on the training set.

Model evaluation was performed independently on both training and test sets. Discrimination was assessed using the sensitivity, specificity, overall accuracy, and area under the receiver operating characteristic curve (AUC). To further evaluate model stability, we performed 5-fold cross-validation with patient-level stratification, ensuring that all aneurysms from the same patient were allocated to the same fold to respect the clustered data structure. The AUC was calculated for each fold, and the mean AUC with standard deviation (SD) was reported.

To facilitate the clinical applications, we further constructed a nomogram to explain the developed prediction model. We used R software (version 4.3.3) for model development.

### 2.5 Statistical analysis

For each potential predictor, we first performed a univariate GEE model with an exchangeable correlation structure to examine the association between the predictor and presence of wall irregular pulsation. The outcome variable was binary (1 = high-risk group n, 0 = low-risk group). Patient ID was included as the clustering variable to account for the non-independence of multiple aneurysms within the same patient. Odds ratios (OR) with 95% confidence intervals (CI) were calculated to quantify the magnitude of association. Variables with a *p*-value < 0.10 in the univariate analysis were considered for inclusion in the multivariate analysis.

Multivariate GEE analysis was performed to identify independent predictors of the presence of wall irregular pulsation while adjusting for potential confounding variables. We adopted an exchangeable working correlation matrix, which assumes a constant correlation between observations within the same cluster (i.e., same patient). The magnitude of clustering was quantified using the intraclass correlation coefficient (ICC). The ICC was estimated as the alpha parameter from the exchangeable GEE model, which represents the constant pairwise correlation among outcomes for aneurysms originating from the same patient. We checked for multicollinearity by examining correlations between continuous variables and variance inflation factors for the final model. All analyses were performed using R software (version 4.3.3).

## 3 Results

### 3.1 Baseline characteristics

A total of 202 patients with 262 UIAs were included in the final analysis. Table 1 shows the baseline characteristics of the study population. There were 64 males and 138 females, with a mean age of 60.0 ± 11.1, ranging from 27 to 85 years. Hypertension was present in half of the patients, while diabetes was less common at 13.9%. Hyperlipidemia was reported in 22.8%, and about 16.8% had a history of smoking. Drinking was noted in 11.4% of cases. Prior subarachnoid hemorrhage was uncommon, affecting only 5.9%.

**Table 1:** Baseline characteristics of the study population.

Variables	Total (*n* = 262)
Number of Patients	202
Sex (Female)	138 (68.3%)
Age (years)	60.0 ± 11.1 [27, 85]
Hypertension	101 (50.0%)
Diabetes	28 (13.9%)
Hyperlipidemia	46 (22.8%)
Smoking	34 (16.8%)
Drinking	23 (11.4%)
Prior subarachnoid hemorrhage	12 (5.9%)

Characteristics of all aneurysms and a comparison between the low- and high-risk aneurysm groups are presented in Table 2. Aneurysms in the high-risk group had larger aneurysm-related dimensions compared to those in the low-risk group: aneurysm size, neck size, perpendicular height, aneurysm maximum height, vessel size, size ratio, aspect ratio, and vessel angle. Compared to the low-risk group, aneurysms in the high-risk group were commonly located in the MCA (12.2% vs. 7.9%) and ACA/PCom/Posterior (44.9% vs. 30.5%), but less frequently located in the ICA (42.9% vs. 61.6%). Daughter sac was markedly more common in the high-risk group (21.4% vs. 4.3%).

**Table 2:** Baseline aneurysm characteristics and comparison between the low- and high-risk group.

Variables*	Total (*n* = 262)	Low-risk group (*n* = 164)	High-risk group (*n* = 98)
Locations			
ICA	143 (54.6%)	101 (61.6%)	42 (42.9%)
MCA	25 (9.5%)	13 (7.9%)	12 (12.2%)
ACA/PCom/Posterior	94 (35.9%)	50 (30.5%)	44 (44.9%)
Daughter sac	28 (10.7%)	7 (4.3%)	21 (21.4%)
Aneurysm size	5.8 ± 3.7	5.2 ± 3.7	6.7 ± 3.5
Neck size	3.9 ± 1.6	3.7 ± 1.7	4.3 ± 1.6
Perpendicular height	3.9 ± 3.0	3.5 ± 3.0	4.6 ± 2.9
Aspect ratio	0.98 ± 0.56	0.9 ± 0.5	1.1 ± 0.7
Maximum height	4.4 ± 3.3	3.8 ± 3.2	5.3 ± 3.3
Vessel size	3.3 ± 0.9	3.4 ± 0.9	3.1 ± 0.8
Size ratio	1.49 ± 1.36	1.2 ± 1.2	1.9 ± 1.5
Aneurysm angle	67.5 ± 19.1	68.7 ± 19.6	65.5 ± 18.2
Vessel angle	20.2 ± 25.1	17.8 ± 24.0	24.1 ± 26.6
Flow angle	96.9 ± 32.6	95.0 ± 31.2	100.1 ± 34.9

* The units of size and angle related variables are mm and degree, respectively.

### 3.2 Univariate and multivariate analyses of predictors of wall irregular pulsation

Among the 262 aneurysms included in the analysis, 152 (58.0%) were from patients with a single aneurysm, 86 (32.8%) from patients with two aneurysms, and 24 (9.2%) from patients with three or four aneurysms. The estimated ICC from the exchangeable GEE model was 0.203 (SE = 0.071), indicating a moderate degree of within-patient correlation.

Table 3 presents the results of univariate and multivariate analyses of predictors for wall irregular pulsation Aneurysm location at ACA/PCom/Posterior (OR: 2.17, 95% CI 1.21–3.88; *p* = 0.0099), presence of daughter sac (OR: 5.15, 95% CI 1.91–13.86; *p* = 0.001), aspect ratio (OR: 1.80, 95% CI 1.09–2.99; *p* = 0.022), and neck size (OR: 1.23, 95% CI 1.02–1.48; *p* = 0.030) were independent risk factors.

**Table 3:** Univariate and multivariate analysis for variables associated with aneurysm rupture risk.

Variables	Univariate analysis		Multivariate analysis	
	OR (95 CI)	*P*	OR (95 CI)	*P*
Age	1.00 (0.97–1.02)	0.811		
Male	1.25 (0.71–2.19)	0.44		
Diabetes	0.87 (0.42–1.78)	0.697		
Hypertension	1.45 (0.86–2.43)	0.163		
Smoking	1.72 (0.87–3.42)	0.121		
Drinking	1.15 (0.52–2.57)	0.728		
Hyperlipidemia	1.36 (0.72–2.58)	0.342		
Prior SAH	1.95 (0.69–5.53)	0.208		
Location				
ICA	References		References	
MCA	2.24 (0.89–5.64)	0.086	2.02 (0.71–5.74)	0.189
ACA/PCom/Posterior	2.07 (1.20–3.56)	0.009	2.17 (1.21–3.88)	0.009
Aneurysm size	1.13 (1.02–1.24)	0.014		
Neck size	1.27 (1.05–1.53)	0.012	1.23 (1.02–1.48)	0.030
Perpendicular height	1.14 (1.02–1.29)	0.027		
Maximum height	1.16 (1.03–1.30)	0.011		
Vessel size	0.67 (0.50–0.89)	0.006		
Aspect ratio	1.95 (1.18–3.22)	0.009	1.80 (1.09–2.99)	0.022
Size ratio	1.57 (1.12–2.19)	0.008		
Vessel angle	1.01 (1.00–1.02)	0.043		
Aneurysm angle	0.99 (0.98–1.00)	0.142		
Flow angle	1.00 (1.00–1.01)	0.195		
Daughter sac	6.33 (2.60–15.45)	<0.001	5.15 (1.91–13.86)	0.001

### 3.3 Development and performance of the predictive model

Baseline characteristics of the training and test datasets are summarized in Table 4. Five clinically and morphologically relevant features—aneurysm location, history of prior SAH, aspect ratio, presence of a daughter sac, and aneurysm neck size—were selected for inclusion in the predictive model. Figure 3 presents the receiver operating characteristic (ROC) curve for the test dataset. The final model demonstrated a sensitivity of 88.5%, specificity of 71.2%, overall accuracy of 76.9%, and an AUC of 0.808 (95% CI, 0.704–0.913). On the independent validation set, the model achieved a sensitivity of 76.9%, specificity of 88.9%, overall accuracy of 81.8%, and an AUC of 0.855 (95% CI, 0.694–1.000). The 5-fold cross-validation yields a mean AUC of 0.699 (SD: 0.066, range: 0.611–0.790). The cross-validation AUC and the range across folds indicate considerable variability.

**Table 4:** Comparison of baseline characteristics between the training and test cohorts.

Variables*	Training cohort (*n* = 210)	Test cohort (*n* = 52)	*P*
Male	64 (30.5%)	12 (23.1%)	0.292
Age	60.3 ± 11.0	59.4 ± 9.5	0.588
Hypertension	100 (47.6%)	27 (51.9%)	0.578
Diabetes	30 (14.3%)	7 (13.5%)	0.879
Hyperlipidemia	45 (21.4%)	10 (19.2%)	0.728
Smoking	35 (16.7%)	6 (11.5%)	0.362
Drinking	24 (11.4%)	3 (5.8%)	0.311
Prior SAH	11 (5.2%)	4 (7.7%)	0.507
Locations			0.480
ICA	112 (53.3%)	31 (59.6%)	
MCA	19 (9.0%)	6 (11.5%)	
ACA/PCom/Posterior	79 (37.6%)	15 (28.8%)	
Daughter sac	20 (9.5%)	8 (15.4%)	0.221
Aneurysm size	5.8 ± 3.7	5.9 ± 3.6	0.822
Neck size	3.9 ± 1.7	4.1 ± 1.6	0.396
Perpendicular height	3.9 ± 3.0	3.8 ± 2.9	0.838
Aspect ratio	0.99 ± 0.57	0.91 ± 0.51	0.347
Aneurysm maximum height	4.4 ± 3.3	4.3 ± 3.2	0.850
Vessel size	3.3 ± 0.9	3.4 ± 0.8	0.309
Size ratio	1.51 ± 1.40	1.39 ± 1.17	0.562
Aneurysm angle	67.4 ± 19.3	68.0 ± 18.2	0.839
Vessel angle	19.3 ± 24.6	23.8 ± 26.9	0.242
Flow angle	95.7 ± 32.0	101.7 ± 34.9	0.236

* The units of size variables and angle are mm and degree, respectively.

**Figure 3:**
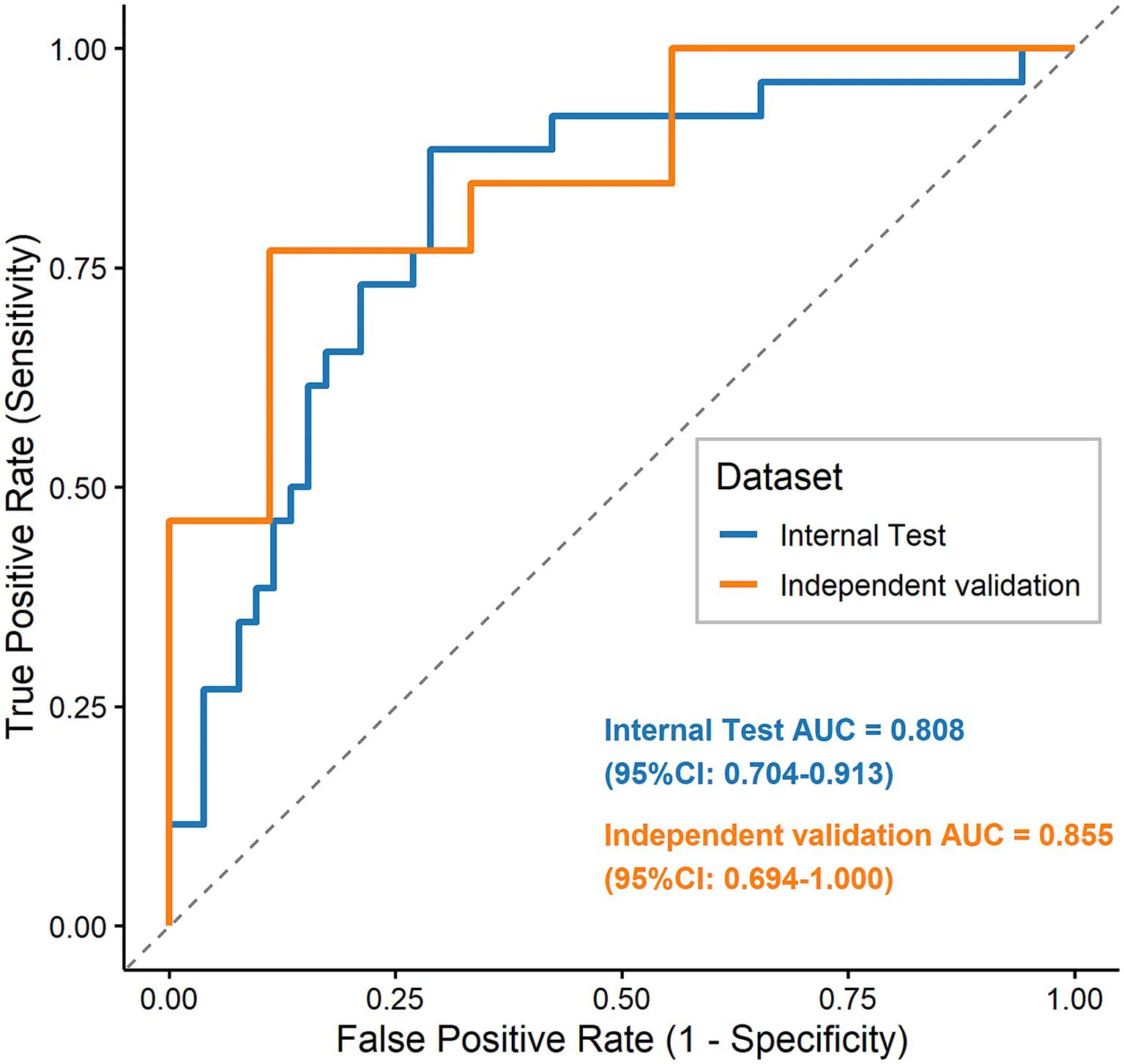
Receiver operator characteristic curve of the developed model in the test dataset.

Figure 4 presents the nomogram providing a visual interpretation of the developed model. The scoring points can be read from the “Point” reference line according to the variable values, including aneurysm location, history of prior SAH, aspect ratio, presence of a daughter sac, and aneurysm neck size. The total points can be obtained by summing the individual variable points. According to the total points, the corresponding predicted risk probability can be read from the bottom “Probability” line.

**Figure 4:**
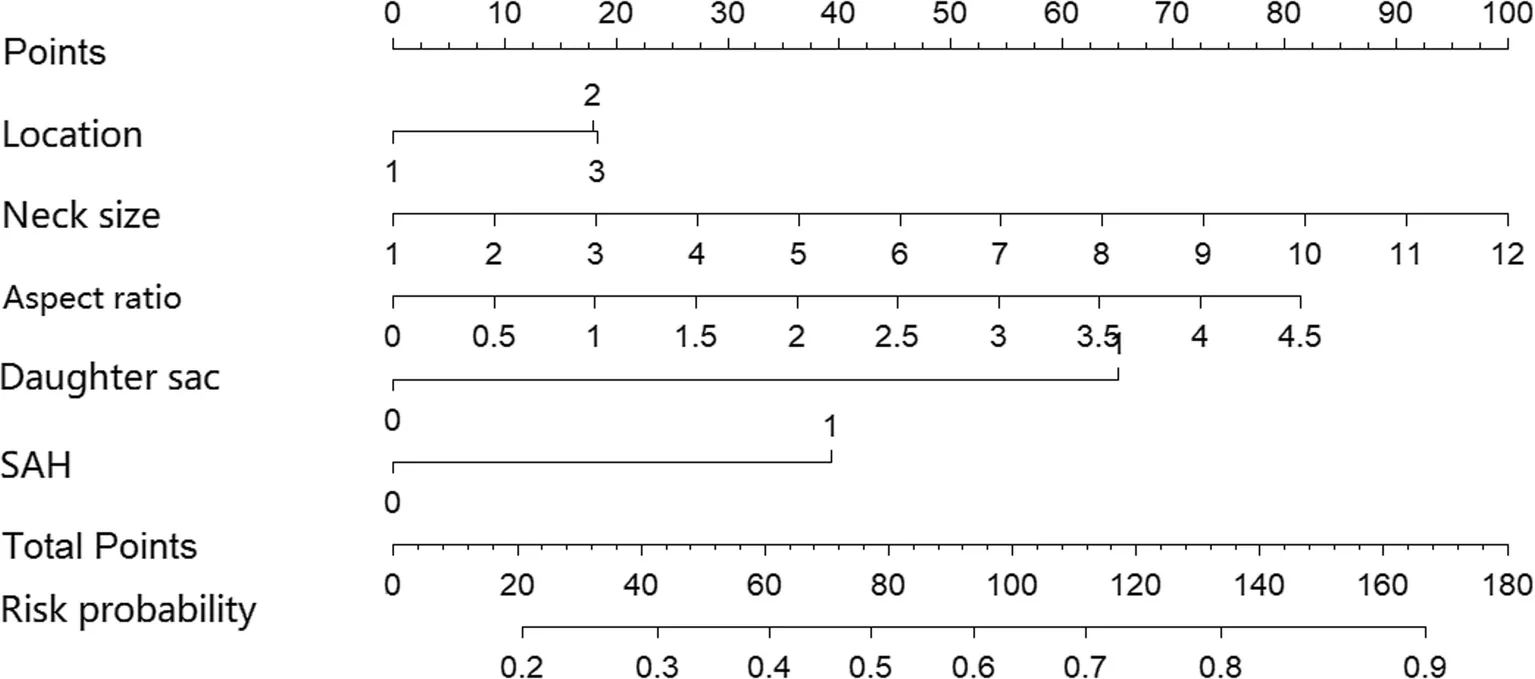
A nomogram for risk stratification of unruptured intracranial aneurysms. Prior SAH: 0, no; 1, yes. Location: 1, internal carotid artery; 2, middle cerebral artery, and 3, anterior cerebral arteries/posterior communicating artery/posterior circulation. Presence of daughter sac: 0, no; 1, yes.

## 4 Discussion

In this study, we used 4D-CTA to identify the wall irregular pulsation and developed a logistic regression-based model using clinical variables to predict wall irregular pulsation in UIAs. Our model demonstrated good predictive performance with an AUC of 0.808 (95% CI, 0.704–0.913) in the test dataset. An interpretable nomogram was subsequently developed to facilitate clinical translation of the prediction model. This model shows potential for future clinical implementation.

The association between aneurysm wall motion and rupture risk has prompted the development of various imaging modalities. Current clinical techniques for wall motion assessment include transcranial power Doppler ultrasonography, phase-contrast MR angiography, 3D rotational angiography, and 4D-CTA—each with distinct technical limitations (16). While power Doppler suffers from acoustic window constraints due to high bone attenuation, phase-contrast MRA faces challenges in clinical adoption due to prolonged acquisition times, inadequate spatial resolution, and lack of standardized blood-velocity values. 3D rotational angiography is the most invasive, while both it and 4D-CTA require contrast administration and exposure to radiation. Nevertheless, 4D-CTA offers practical advantages, including widespread scanner availability, rapid acquisition, and superior spatial resolution (16, 17). Clinical validations have established irregular pulsation patterns on 4D-CTA as reliable biomarkers for stratifying rupture risk in UIAs (5–7). In this study, we adopted 4D-CTA as an intermediary tool to categorize UIAs into low- and high-risk groups.

In this study, five parameters were incorporated into the prediction model: Prior SAH, aneurysm location, aspect ratio, presence of daughter sac, and aneurysm neck size. As wall irregular pulsation may represent a biomechanical correlate of aneurysm instability, it is plausible that these same risk factors also serve as predictors of this imaging-derived phenotype. Previous SAH is a core component of both the PHASES and ELAPSS scoring systems (14, 15), and our finding that it also predicts irregular pulsation extends this association from rupture to a pre-clinical hemodynamic indicator. Aneurysm location has been well-documented to demonstrate an association with aneurysm rupture or growth (14, 15, 18). In our study, location was significantly associated with irregular pulsation in both univariate and multivariable analysis, confirming its independent predictive value. Importantly, morphologic features including aspect ratio and daughter sac has been robustly linked to elevated rupture or growth risk (15, 18–21). Both of the two morphologic features also emerged as independent predictors in the multivariable model, suggesting that morphology provides incremental prognostic information beyond location alone (13, 22, 23). The relationship between location and morphology may be understood as complementary: location reflects the anatomical hemodynamic environment, whereas morphology captures the aneurysm’s geometric response to these hemodynamic forces.

Irregular pulsation detected on 4D-CTA has emerged as a promising imaging marker of aneurysm wall instability. Previoius studies (6, 7, 13) have demonstrated that irregular pulsation is independently associated with aneurysm rupture, symptomatic IAs, and aneurysm wall enhancement on vessel wall MRI. Moreover, irregular pulsation has been linked to conventional rupture risk factors and increased estimated rupture risk (23), and 4D-CTA-derived wall dynamics have shown utility in predicting aneurysm growth and longitudinal size change (24). These findings suggest that irregular pulsation reflects underlying hemodynamic and wall abnormalities that precede overt rupture, supporting its role as a pre-clinical indicator of aneurysm vulnerability.

Recently reported computational models, including random forest, support vector machine, artificial neural network, lasso regression, and deep learning architectures, may enhance the prediction of rupture risk in intracranial aneurysms (25–29). A comprehensive meta-analysis (30) encompassing 36 studies with 22,462 patients revealed that these computational approaches, irrespective of specific implementations, achieved comparable sensitivity (0.743 versus 0.771), superior specificity (0.763 versus 0.507), and higher AUC values (0.84 versus 0.64) compared to the PHASES score. Notably, deep learning demonstrated enhanced predictive capabilities in external validation groups, with sensitivities and specificities reaching 0.792 and 0.788, respectively (30). In contrast to these complex “black-box” algorithms, our model addresses this gap by developing an interpretable nomogram based on conventional logistic regression that directly predicts irregular pulsation, thereby providing a practical tool for early identification of aneurysm wall instability before the onset of actual rupture. By combining morphological and clinical features, the nomogram achieves both predictive performance and visual interpretability, enhancing its clinical applicability.

This study has several limitations. First, this was a post-hoc analysis of a prospective study, which inherently carries the risks of selection bias. Second, we defined the risk of rupture based on the 4D-CTA images, and an accurate assessment of the risk of rupture is needed for validation through long-term follow-up of UIAs. Third, the statistical power may be constrained by the limited sample size. Fourth, a potential limitation is that the 5-fold cross-validation AUCs showed considerable variability (range: 0.611–0.790), and the mean AUC (0.699) was lower than the test-set AUC (0.808). This discrepancy suggests that model performance may be sensitive to sample composition, and further validation in larger, independent external cohorts is needed to establish the model’s robustness and generalizability.

In conclusion, we developed a logistic regression-based model incorporating both clinical and morphologic variables to predict the presence of wall irregular pulsation. To facilitate clinical applications, an intuitive nomogram was constructed to visualize the predictive value of the developed model.

## Data Availability

The raw data supporting the conclusions of this article will be made available by the authors, without undue reservation.
