# Supplementary material for: Development of a nomogram for predicting wall irregular pulsation and risk stratification in intracranial aneurysms
Source: Front Neurol. 2026 Sep 28;17:1913037. doi: 10.3389/fneur.2026.1913037 (PMC13654754; doi:10.3389/fneur.2026.1913037)
Supplement: Supplementary file 1 [file Table_1.docx]

Supplementary Table 1. Comparison of baseline characteristics between development and validation cohorts

| Variables | Development cohort (n = 262) | Validation cohort (n = 22) | *P* value |
| --- | --- | --- | --- |
| Patient-level variables | | | |
| Age (years) | 60.0 ± 11.1 | 62.6 ± 12.4 | 0.156 |
| Male sex | 64 (31.7%) | 6 (30.0%) | >0.999 |
| Hypertension | 101 (50.0%) | 6 (30.0%) | 0.104 |
| Diabetes | 28 (13.9%) | 0 (0.0%) | 0.085 |
| Hyperlipidemia | 46 (22.8%) | 0 (0.0%) | 0.017 |
| Smoking | 34 (16.8%) | 0 (0.0%) | 0.049 |
| Drinking | 23 (11.4%) | 0 (0.0%) | 0.238 |
|  | | | |
| Aneurysm-level variables | | | |
| Prior subarachnoid hemorrhage | 12 (5.9%) | 0 (0.0%) | 0.247 |
| Daughter sac | 28 (10.7%) | 4 (18.2%) | 0.290 |
| Location  ICA  MCA  ACA/PCom/Posterior | 143 (54.6%)  25 (9.5%)  94 (35.9%) | 12 (54.5%)  3 (13.6%)  7 (31.8%) | 0.061 |
| Aneurysm size (mm) | 5.8 ± 3.7 | 7.8 ± 4.0 | 0.007 |
| Neck width (mm) | 3.9 ± 1.6 | 4.5 ± 2.7 | 0.516 |
| Perpendicular height (mm) | 3.9 ± 3.0 | 5.4 ± 3.2 | 0.005 |
| Aspect ratio | 0.98 ± 0.56 | 1.43 ± 0.85 | 0.003 |
| Maximum height (mm) | 4.4 ± 3.3 | 7.3 ± 3.8 | <0.001 |
| Vessel size (mm) | 3.3 ± 0.9 | 2.4 ± 0.6 | <0.001 |
| Size ratio | 1.49 ± 1.36 | 1.83 ± 1.01 | 0.007 |
| Aneurysm angle (°) | 67.5 ± 19.1 | 84.9 ± 25.9 | 0.009 |
| Vessel angle (°) | 20.2 ± 25.1 | 43.7 ± 39.5 | <0.001 |
| Flow angle (°) | 96.9 ± 32.6 | 120.6 ± 33.9 | 0.002 |
